# Synthesis and crystal structure of a new pyridinium bromide salt: 4-methyl-1-(3-phen­oxy­prop­yl)pyridinium bromide

**DOI:** 10.1107/S2056989017015481

**Published:** 2017-11-03

**Authors:** Musa A. Said, Mohamed R. Aouad, David L. Hughes, Meshal A. Almehmadi, Mouslim Messali

**Affiliations:** a Department of Chemistry, Taibah University, 30002, Al-Madina Al-Mounawara, Saudi Arabia; b Laboratoire de Chimie & Electrochimie des Complexes Métalliques (LCECM), USTO-MB, University of Sciences and Technology Mohamed Boudiaf, BP 1505 Oran, El M’nouar, Algeria; c School of Chemistry, University of East Anglia, University Plain, Norwich NR4 7TJ, United Kingdom

**Keywords:** crystal structure, ionic liquids, pyridinium salts, halide, hydrogen bonding, π–π inter­actions

## Abstract

In the cation of the title mol­ecular salt, C_15_H_18_NO^+^·Br^−^, the pyridinium and phenyl rings are inclined to one another by 11.80 (8)°. In the crystal, the Br^−^ anion is linked to the cation by a C—H⋯Br hydrogen bond. The cations stack along the *b*-axis direction and are linked by further C—H⋯Br inter­actions, and offset π–π inter­actions [inter­centroid distances = 3.5733 (19) and 3.8457 (19) Å], forming slabs parallel to the *ab* plane. The effects of the C—H⋯*X* ^−^ inter­action on the NMR signals of the *ortho-* and *meta-*pyridinium protons in a series of related ionic liquids, *viz*. 4-methyl-1-(4-phen­oxy­but­yl)pyridin-1-ium salts, are reported and discussed.

## Abstract 2

The simple synthesis and crystal structure of a new pyridinium bromide salt, 4-methyl-1-(3-phen­oxy prop­yl)pyridinium bromide, are reported. The C–H⋯Br^−^ inter­actions have an effect on the NMR signals of the *ortho-* and *meta-*pyridinium protons.

## Chemical context   

In the last two decades, ionic liquids (ILs) have gained considerable inter­est as excellent alternatives to volatile organic compounds (VOCs) because of their unusual range of properties such as negligible vapour pressure, excellent thermal stability in a wide temperature range, no flammability, high ionic conductivity and solvation ability (Davis, 2004 ▸).

A wide range of applications using ionic liquids has been reported in many areas such as solvents in organic synthesis (Wang *et al.*, 2007 ▸), media for electrodeposition of metals (Endres, 2002 ▸), corrosion inhibitors (Ibrahim *et al.*, 2011 ▸), electrolytes for electrochemical devices such as batteries (Brennecke & Maginn, 2001 ▸), catalysts (Shi *et al.* 2004 ▸), in fuel cells (De Souza *et al.*, 2006 ▸), in polymer science (Kubisa, 2004 ▸), and in dye-sensitized solar cells (Kawano *et al.*, 2004 ▸).

In view of the above mentioned, and of our ongoing research inter­est in the synthesis of ionic liquids (Messali, 2016 ▸, 2015 ▸; Messali *et al.*, 2014 ▸), we present in this study the preparation and the crystal structure of the novel title pyridinium halide salt, 4-methyl-1-(3-phen­oxy­prop­yl)pyridinium bromide.
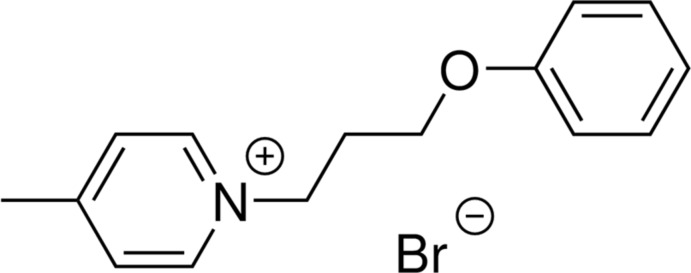


## Structural commentary   

**Figure 1:**
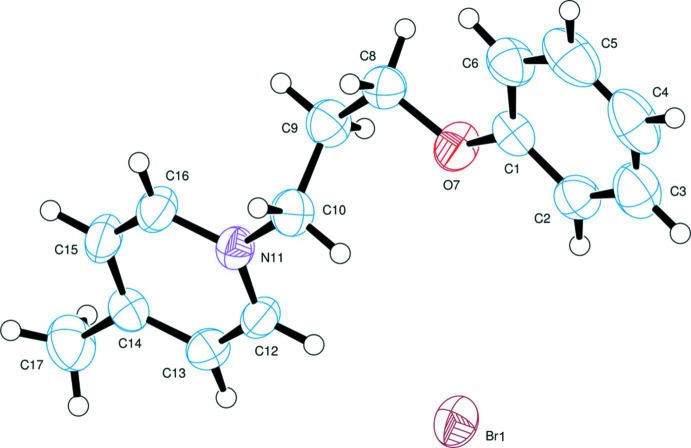
The mol­ecular structure of the component ions of the title salt, indicating the atom-numbering scheme. Displacement ellipsoids are drawn at the 50% probability level.

**Figure 2:**
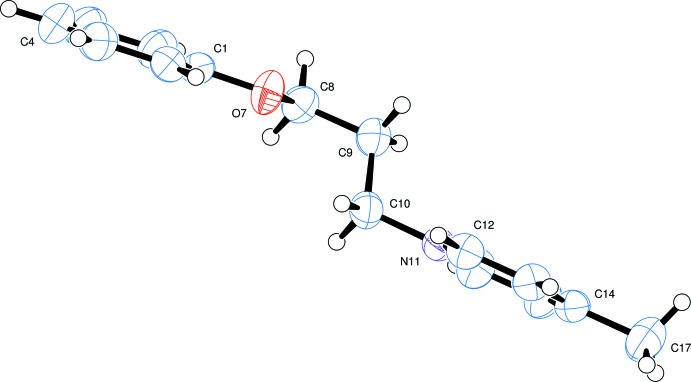
A view of the cation showing the step formation about bond C9—C10 and the approximately parallel ring planes.

The mol­ecular structure of the title pyridinium bromide salt is illustrated in Fig. 1 ▸. There is a weak intra­molecular C—H⋯O contact present, with an H⋯O distance of 2.52 Å and a C—H⋯O angle of only 100° (see Table 1 ▸). The cation consists of two planar groups, a pyridinium ring (N11/C12–C16) and a phenyl group (C1–C6); atom N11 has the expected planar–trigonal conformation. The two aromatic rings are inclined to one another by 11.80 (8)° and there is a step of *ca* 1.35 Å between the two groups along the C9—C10 bond, see Fig. 2 ▸. The C1—O7, C8—C9, C10—N11 and C14—C17 bonds are roughly parallel, so that the two aromatic groups are at opposite ends of an approximately linear cation. This is in contrast to the alignment found in 1-[2-(5-nitro-1*H*-indol-2-yl)phen­yl]methyl­pyridinium chloride where the cation is U-shaped with the pyridinium ring lying over the pyrrolo ring of the indole moiety (Bremner *et al.*, 2011 ▸), possibly as a result of electronic inter­actions between the two rings.

## Supra­molecular features   

In the crystal, the bromide anion is linked to the cation by a C10—H10*B*⋯Br1^i^ hydrogen bond (Table 1 ▸). The anion is surrounded by three other cations with the most significant C-*–*H⋯Br short contacts varying from *ca* 3.07 to 3.11 Å (Table 1 ▸). The bromide ions are aligned approximately in the planes of the aromatic rings, which is similar to the arrangement found in *N*-benzyl­pyridinium bromide (Anders *et al.*, 1990 ▸), and in contrast to those in a series of *N*-(penta­fluoro­benz­yl)pyridinium salts where the anion faces the aromatic rings with formation of anion–π inter­actions (Giese *et al.*, 2014 ▸).

**Figure 3:**
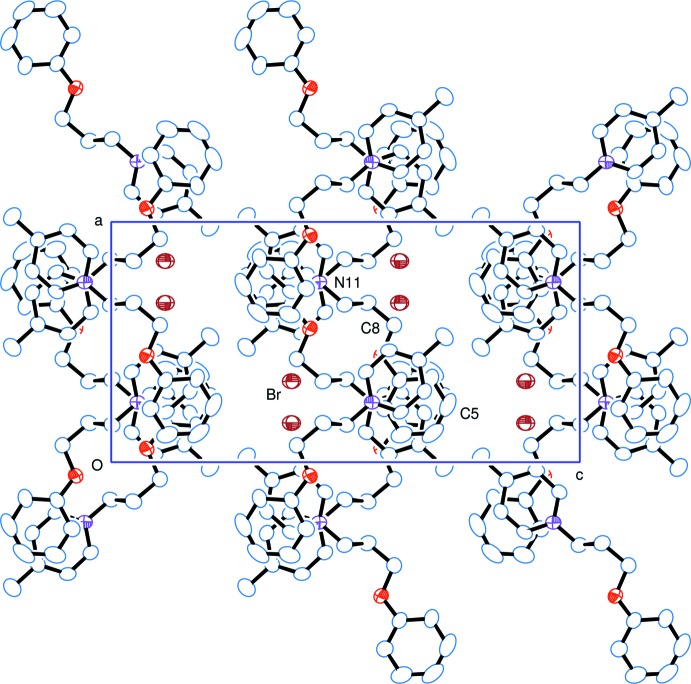
Crystal packing viewed along the *b* axis, showing the stacking of the phenyl and pyridinium groups along that axis.

The cations stack head-to-tail, in pairs about centres of symmetry, along the *b-*axis direction with the aromatic rings being inclined slightly to one another [α = 11.80 (8)° within a pair and 7.52 (16)° between pairs]. As shown in Fig. 3 ▸, the pairs are linked by offset π–π inter­actions, forming slabs parallel to (001): *Cg*1⋯*Cg*2^iii^ = 3.8457 (19) Å within a pair, and *Cg*1⋯*Cg*2^iv^ = 3.5733 (19) Å between pairs; *Cg*1 and *Cg*2 are the centroids of rings C1—C6 and N11/C12–C16, respect­ively; symmetry codes: (iii) 1 − *x*, 1 − *y*, 1 − *z*; (iv) *x* − 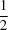, 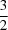 − *y*, 1 − *z*.

## C—H⋯anion inter­actions in the ^1^H NMR spectrum   

**Figure 4:**
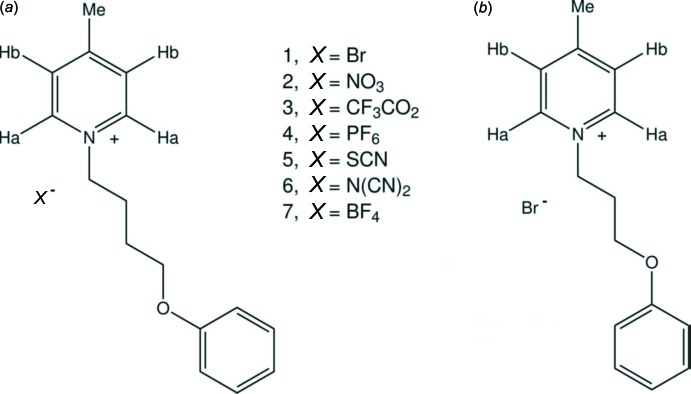
A series of ionic liquids: (*a*) 4-methyl-1-(4-phen­oxy­but­yl)pyridin-1-ium salts with various counter-anions; (*b*) this study: 4-methyl-1-(3-phen­oxy­prop­yl)pyridinium bromide.

The C—H⋯anion inter­actions are clearly manifested in the ^1^H NMR spectrum (see Section 5. *Synthesis and crystallization*). Such an effect has previously been shown by a solution study of the C—H⋯Br inter­action on the signals of the *ortho*- and *meta*-pyridinium protons in the ^1^H NMR spectra of a series of *N*-(penta­fluoro­benz­yl)pyridinium salts (Giese *et al.*, 2014 ▸). The present study in D_2_O solvent involves only the pyridinium protons (H*a* and H*b*) of a series of 4-methyl-1-(4-phen­oxy­but­yl)pyridin-1-ium *X* ^−^ ionic liquids, and the title compound, shown in Fig. 4 ▸. The results, given in Table 2 ▸, reveal significant shifts for the hydrogen atom H*a* in various pyridinium salts, whereas hydrogen atom H*b* is only slightly affected by the different counter-anions (Messali, 2015 ▸); *viz.* the study reveals a range of 0.75 p.p.m. for the signals of the *ortho*-pyridinium protons (H*a*) and a shorter range of 0.29 p.p.m. for *meta*-pyridinium protons (H*b*). The determination of the causes behind this variation remains a challenging task for our research group.

## Synthesis and crystallization   

**Figure 5:**
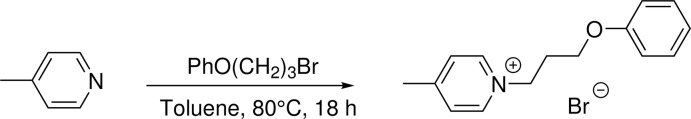
Synthesis of the title compound.

The synthesis of the title compound is illustrated in Fig. 5 ▸. To a solution of 1 g of 4-picoline (10.7 mmol) in 20 ml of toluene, were added 2.53 g of (3-bromo­prop­oxy)benzene (118 mmol) at room temperature, followed by stirring at 355 K for 18 h. The completion of the reaction was marked by the separation of a solid from the initially obtained clear and homogeneous mixture of the starting materials. The product was isolated by filtration to remove the unreacted starting materials and solvent. Subsequently, the title picolinium salt was washed with ethyl acetate. The product was finally dried at reduced pressure to remove all volatile organic compounds. The title compound was obtained as a white solid. Colourless prismatic crystals were obtained by slow evaporation of a solution in di­chloro­methane.

Spectroscopic and analytical data: ^1^H NMR (D_2_O, 400 MHz): δ = 2.34 (*quint*, *J* = 7.6 Hz, 2H), 2.50 (*s*, 3H), 3.98 (*t*, *J* = 7.6 Hz, 2H), 4.60 (*t*, *J* = 7.6 Hz, 2H), 6.72 (*d*, 2Ar–H), 6.91 (*t*, 1Ar–H), 7.21 (*t*, 2Ar–H), 7.66 (*d*, 2Ar–H), 8.48 (*d*, 2Ar–H); ^13^C NMR (D_2_O, 100 MHz,): δ = 21.2 (CH_3_), 29.4 (CH_2_), 58.5 (CH_2_), 64.5 (CH_2_), 114.4 (CH), 121.5 (CH), 128.5 (CH), 129.8 (CH), 143.2 (CH), 157.4 (C), 160.1 (C); IR (KBr) ν_max_ 3132 (C—H Ar), 1600–1470 (C=C), 1167(C—N), 1078 (C—O) cm^−1^; LCMS (*M* ^+^)–Br^−^ 228.1 found for C_15_H_18_NO^+^. Elemental analysis for C_15_H_18_BrNO (308.21); calculated C 58.45, H 5.89, N 4.54%. Found: C 58.51, H 5.82, N 4.49%.

## Refinement   

Crystal data, data collection and structure refinement details are summarized in Table 3 ▸. The H atoms were included in idealized positions and treated as riding atoms: C—H = 0.93–0.97 Å with *U*_iso_(H) = 1.5*U*_eq_(C-meth­yl) and = 1.2*U*_eq_(C) for other H atoms.

## Supplementary Material

Crystal structure: contains datablock(s) I, Global. DOI: 10.1107/S2056989017015481/su5395sup1.cif

Structure factors: contains datablock(s) I. DOI: 10.1107/S2056989017015481/su5395Isup2.hkl

Supporting information file. DOI: 10.1107/S2056989017015481/su5395Isup3.cdx

Supporting information file. DOI: 10.1107/S2056989017015481/su5395Isup4.cml

CCDC reference: 1581713

Additional supporting information:  crystallographic information; 3D view; checkCIF report

## Figures and Tables

**Table 1:** Hydrogen-bond geometry (Å, °)

*D*—H⋯*A*	*D*—H	H⋯*A*	*D*⋯*A*	*D*—H⋯*A*
C10—H10*A*⋯O7	0.97	2.52	2.850 (3)	100
C10—H10*B*⋯Br1^i^	0.97	2.89	3.735 (3)	146
C10—H10*A*⋯Br1	0.97	3.11	4.043 (3)	163
C12—H12⋯Br1	0.93	3.08	3.940 (3)	154
C15—H15⋯Br1^ii^	0.93	3.07	3.931 (3)	155
C17—H17*B*⋯Br1^ii^	0.96	3.08	3.956 (4)	152

Symmetry codes: (i) 
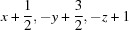
; (ii) 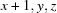.

**Table 2:** ^1^H NMR chemical shifts (D_2_O, δ p.p.m.) for the pyridinium hydrogen atoms (H*a* and H*b*) of a series of ionic liquids (1–7)* and the title salt

Ionic liquid	Anion	Chemical shift for H*a*	Chemical shift for H*b*
1	Br^−^	*d*, 9.23	*d*, 7.67
2	NO_3_^−^	*d*, 9.22	*d*, 7.66
3	CF_3_CO_2_^−^	*d*, 9.09	*d*, 7.65
4	PF_6_^−^	*d*, 8.94	*d*, 7.94
5	SCN^−^	*d*, 8.82	*d*, 7.80
6	N(CN)_2_^−^	*d*, 8.91	*d*, 7.75
7	BF_4_^−^	*d*, 8.60	*d*, 7.69
This study	Br^−^	*d*, 8.48	*d*, 7.66

*Messali (2015 ▸).

**Table 3:** Experimental details

Crystal data	
Chemical formula	C_15_H_18_NO^+^·Br^−^
*M*_r_	308.21
Crystal system, space group	Orthorhombic, *P* *b* *c* *a*
Temperature (K)	295
*a*, *b*, *c* (Å)	10.3615 (3), 13.8916 (6), 20.2121 (8)
*V* (Å^3^)	2909.29 (19)
*Z*	8
Radiation type	Mo *K*α
μ (mm^−1^)	2.82
Crystal size (mm)	0.60 × 0.19 × 0.10

Data collection	
Diffractometer	Oxford Diffraction Xcalibur 3/Sapphire3 CCD
Absorption correction	Multi-scan (*CrysAlis PRO*; Agilent, 2014 ▸)
*T*_min_, *T*_max_	0.529, 1.000
No. of measured, independent and observed [*I* > 2σ(*I*)] reflections	38980, 2560, 2212
*R*_int_	0.048
(sin θ/λ)_max_ (Å^−1^)	0.595

Refinement	
*R*[*F* ^2^ > 2σ(*F* ^2^)], *wR*(*F* ^2^), *S*	0.045, 0.087, 1.20
No. of reflections	2560
No. of parameters	164
H-atom treatment	H-atom parameters constrained
Δρ_max_, Δρ_min_ (e Å^−3^)	0.27, −0.25

Computer programs: *CrysAlis PRO* (Agilent, 2014 ▸), *SHELXS97* (Sheldrick, 2008 ▸), *SHELXL2014* (Sheldrick, 2015 ▸) and *ORTEP-3 for Windows* and *WinGX* (Farrugia, 2012 ▸).

## supplementary crystallographic information

### Crystal data

**Table:**

C_15_H_18_NO^+^·Br^−^	*D*_x_ = 1.407 Mg m^−^^3^
*M**_r_* = 308.21	Mo *K*α radiation, λ = 0.71073 Å
Orthorhombic, *P**b**c**a*	Cell parameters from 5457 reflections
*a* = 10.3615 (3) Å	θ = 3.2–25.6°
*b* = 13.8916 (6) Å	µ = 2.82 mm^−^^1^
*c* = 20.2121 (8) Å	*T* = 295 K
*V* = 2909.29 (19) Å^3^	Prism, colourless
*Z* = 8	0.60 × 0.19 × 0.10 mm
*F*(000) = 1264	

### Data collection

**Table:**

Oxford Diffraction Xcalibur 3/Sapphire3 CCD diffractometer	2560 independent reflections
Radiation source: Enhance (Mo) X-ray Source	2212 reflections with *I* > 2σ(*I*)
Graphite monochromator	*R*_int_ = 0.048
Detector resolution: 16.0050 pixels mm^-1^	θ_max_ = 25.0°, θ_min_ = 3.6°
Thin slice φ and ω scans	*h* = −12→12
Absorption correction: multi-scan (CrysAlis Pro; Agilent, 2014)	*k* = −16→16
*T*_min_ = 0.529, *T*_max_ = 1.000	*l* = −24→24
38980 measured reflections	

### Refinement

**Table:**

Refinement on *F*^2^	Primary atom site location: structure-invariant direct methods
Least-squares matrix: full	Secondary atom site location: difference Fourier map
*R*[*F*^2^ > 2σ(*F*^2^)] = 0.045	Hydrogen site location: inferred from neighbouring sites
*wR*(*F*^2^) = 0.087	H-atom parameters constrained
*S* = 1.20	*w* = 1/[σ^2^(*F*_o_^2^) + (0.0313*P*)^2^ + 1.5286*P*] where *P* = (*F*_o_^2^ + 2*F*_c_^2^)/3
2560 reflections	(Δ/σ)_max_ = 0.001
164 parameters	Δρ_max_ = 0.27 e Å^−^^3^
0 restraints	Δρ_min_ = −0.25 e Å^−^^3^

### Special details

**Table:**

Geometry. All esds (except the esd in the dihedral angle between two l.s. planes) are estimated using the full covariance matrix. The cell esds are taken into account individually in the estimation of esds in distances, angles and torsion angles; correlations between esds in cell parameters are only used when they are defined by crystal symmetry. An approximate (isotropic) treatment of cell esds is used for estimating esds involving l.s. planes.

### Fractional atomic coordinates and isotropic or equivalent isotropic displacement parameters (Å^2^)

**Table:**

	*x*	*y*	*z*	*U*_iso_*/*U*_eq_	
Br1	0.33702 (3)	0.68529 (3)	0.38455 (2)	0.05802 (14)	
C1	0.3412 (3)	0.6101 (2)	0.61560 (14)	0.0449 (7)	
C2	0.2231 (3)	0.6169 (2)	0.58402 (17)	0.0497 (8)	
H2	0.2174	0.6070	0.5386	0.060*	
C3	0.1146 (4)	0.6382 (3)	0.6199 (2)	0.0665 (10)	
H3	0.0352	0.6421	0.5986	0.080*	
C4	0.1213 (4)	0.6540 (3)	0.6864 (2)	0.0768 (12)	
H4	0.0471	0.6694	0.7101	0.092*	
C5	0.2377 (4)	0.6470 (3)	0.71827 (19)	0.0756 (11)	
H5	0.2422	0.6578	0.7636	0.091*	
C6	0.3494 (3)	0.6241 (3)	0.68326 (16)	0.0567 (9)	
H6	0.4281	0.6182	0.7050	0.068*	
O7	0.44380 (18)	0.58949 (16)	0.57589 (10)	0.0516 (6)	
C8	0.5707 (3)	0.5894 (2)	0.60379 (14)	0.0487 (8)	
H8A	0.5792	0.5381	0.6360	0.058*	
H8B	0.5880	0.6503	0.6256	0.058*	
C9	0.6631 (3)	0.5744 (2)	0.54734 (16)	0.0514 (8)	
H9A	0.6427	0.5145	0.5250	0.062*	
H9B	0.7504	0.5696	0.5644	0.062*	
C10	0.6554 (3)	0.6566 (2)	0.49855 (15)	0.0478 (7)	
H10A	0.5687	0.6602	0.4806	0.057*	
H10B	0.6732	0.7167	0.5213	0.057*	
N11	0.7491 (2)	0.64405 (17)	0.44343 (11)	0.0400 (6)	
C12	0.7086 (3)	0.6251 (2)	0.38199 (15)	0.0440 (7)	
H12	0.6206	0.6203	0.3734	0.053*	
C13	0.7952 (3)	0.6126 (2)	0.33169 (15)	0.0469 (7)	
H13	0.7657	0.5996	0.2892	0.056*	
C14	0.9257 (3)	0.6192 (2)	0.34333 (15)	0.0439 (7)	
C15	0.9652 (3)	0.6400 (2)	0.40727 (15)	0.0500 (8)	
H15	1.0527	0.6449	0.4169	0.060*	
C16	0.8761 (3)	0.6533 (2)	0.45620 (16)	0.0493 (8)	
H16	0.9033	0.6688	0.4987	0.059*	
C17	1.0219 (3)	0.6050 (3)	0.28885 (17)	0.0680 (10)	
H17A	1.0431	0.5379	0.2854	0.102*	
H17B	1.0986	0.6411	0.2984	0.102*	
H17C	0.9856	0.6268	0.2478	0.102*	

### Atomic displacement parameters (Å^2^)

**Table:**

	*U*^11^	*U*^22^	*U*^33^	*U*^12^	*U*^13^	*U*^23^
Br1	0.0428 (2)	0.0664 (2)	0.0649 (2)	−0.00036 (16)	0.00019 (15)	−0.00230 (18)
C1	0.0509 (17)	0.0394 (16)	0.0444 (17)	−0.0034 (14)	0.0090 (15)	0.0015 (14)
C2	0.0513 (18)	0.0434 (19)	0.0544 (18)	0.0051 (15)	0.0028 (16)	0.0044 (15)
C3	0.0530 (19)	0.057 (2)	0.089 (3)	0.0066 (18)	0.014 (2)	0.012 (2)
C4	0.073 (3)	0.068 (3)	0.089 (3)	0.002 (2)	0.043 (2)	0.005 (2)
C5	0.104 (3)	0.069 (3)	0.053 (2)	−0.011 (2)	0.030 (2)	−0.0030 (19)
C6	0.066 (2)	0.062 (2)	0.0421 (18)	−0.0088 (17)	0.0079 (16)	−0.0033 (16)
O7	0.0421 (11)	0.0718 (16)	0.0410 (11)	0.0003 (10)	0.0042 (10)	−0.0120 (11)
C8	0.0472 (17)	0.054 (2)	0.0446 (17)	−0.0009 (15)	−0.0007 (14)	0.0019 (15)
C9	0.0454 (17)	0.0531 (19)	0.0557 (19)	0.0045 (15)	0.0050 (15)	0.0003 (16)
C10	0.0431 (16)	0.0490 (18)	0.0513 (18)	0.0030 (14)	0.0041 (14)	−0.0031 (15)
N11	0.0347 (12)	0.0386 (14)	0.0468 (14)	0.0002 (10)	0.0003 (11)	−0.0012 (11)
C12	0.0374 (15)	0.0456 (18)	0.0489 (18)	−0.0023 (13)	−0.0103 (14)	0.0005 (15)
C13	0.0490 (17)	0.0508 (19)	0.0407 (16)	−0.0033 (15)	−0.0055 (14)	−0.0008 (14)
C14	0.0466 (16)	0.0400 (17)	0.0452 (17)	−0.0042 (14)	0.0036 (14)	0.0014 (14)
C15	0.0324 (15)	0.062 (2)	0.0554 (19)	−0.0070 (15)	−0.0019 (14)	−0.0028 (16)
C16	0.0410 (16)	0.061 (2)	0.0462 (18)	−0.0061 (15)	−0.0059 (14)	−0.0058 (16)
C17	0.062 (2)	0.082 (3)	0.059 (2)	−0.007 (2)	0.0151 (18)	−0.0050 (19)

### Geometric parameters (Å, º)

**Table:**

C1—O7	1.362 (3)	C9—H9B	0.9700
C1—C2	1.383 (4)	C10—N11	1.488 (4)
C1—C6	1.384 (4)	C10—H10A	0.9700
C2—C3	1.371 (5)	C10—H10B	0.9700
C2—H2	0.9300	N11—C12	1.337 (3)
C3—C4	1.364 (5)	N11—C16	1.347 (4)
C3—H3	0.9300	C12—C13	1.368 (4)
C4—C5	1.371 (6)	C12—H12	0.9300
C4—H4	0.9300	C13—C14	1.375 (4)
C5—C6	1.393 (5)	C13—H13	0.9300
C5—H5	0.9300	C14—C15	1.386 (4)
C6—H6	0.9300	C14—C17	1.498 (4)
O7—C8	1.431 (3)	C15—C16	1.365 (4)
C8—C9	1.505 (4)	C15—H15	0.9300
C8—H8A	0.9700	C16—H16	0.9300
C8—H8B	0.9700	C17—H17A	0.9600
C9—C10	1.511 (4)	C17—H17B	0.9600
C9—H9A	0.9700	C17—H17C	0.9600
			
O7—C1—C2	115.6 (3)	N11—C10—C9	111.4 (2)
O7—C1—C6	124.3 (3)	N11—C10—H10A	109.3
C2—C1—C6	120.0 (3)	C9—C10—H10A	109.3
C3—C2—C1	119.8 (3)	N11—C10—H10B	109.3
C3—C2—H2	120.1	C9—C10—H10B	109.3
C1—C2—H2	120.1	H10A—C10—H10B	108.0
C4—C3—C2	120.9 (4)	C12—N11—C16	120.2 (2)
C4—C3—H3	119.5	C12—N11—C10	120.9 (2)
C2—C3—H3	119.5	C16—N11—C10	118.9 (2)
C3—C4—C5	119.8 (4)	N11—C12—C13	120.6 (3)
C3—C4—H4	120.1	N11—C12—H12	119.7
C5—C4—H4	120.1	C13—C12—H12	119.7
C4—C5—C6	120.6 (3)	C12—C13—C14	120.7 (3)
C4—C5—H5	119.7	C12—C13—H13	119.7
C6—C5—H5	119.7	C14—C13—H13	119.7
C1—C6—C5	118.9 (3)	C13—C14—C15	117.6 (3)
C1—C6—H6	120.6	C13—C14—C17	121.3 (3)
C5—C6—H6	120.6	C15—C14—C17	121.1 (3)
C1—O7—C8	119.0 (2)	C16—C15—C14	120.3 (3)
O7—C8—C9	106.6 (2)	C16—C15—H15	119.9
O7—C8—H8A	110.4	C14—C15—H15	119.9
C9—C8—H8A	110.4	N11—C16—C15	120.6 (3)
O7—C8—H8B	110.4	N11—C16—H16	119.7
C9—C8—H8B	110.4	C15—C16—H16	119.7
H8A—C8—H8B	108.6	C14—C17—H17A	109.5
C8—C9—C10	110.9 (3)	C14—C17—H17B	109.5
C8—C9—H9A	109.5	H17A—C17—H17B	109.5
C10—C9—H9A	109.5	C14—C17—H17C	109.5
C8—C9—H9B	109.5	H17A—C17—H17C	109.5
C10—C9—H9B	109.5	H17B—C17—H17C	109.5
H9A—C9—H9B	108.1		
			
O7—C1—C2—C3	−179.4 (3)	C9—C10—N11—C12	111.0 (3)
C6—C1—C2—C3	0.5 (5)	C9—C10—N11—C16	−69.8 (3)
C1—C2—C3—C4	0.7 (5)	C16—N11—C12—C13	1.5 (4)
C2—C3—C4—C5	−1.0 (6)	C10—N11—C12—C13	−179.3 (3)
C3—C4—C5—C6	0.0 (6)	N11—C12—C13—C14	0.1 (5)
O7—C1—C6—C5	178.5 (3)	C12—C13—C14—C15	−0.8 (5)
C2—C1—C6—C5	−1.4 (5)	C12—C13—C14—C17	179.5 (3)
C4—C5—C6—C1	1.2 (5)	C13—C14—C15—C16	0.0 (5)
C2—C1—O7—C8	174.7 (3)	C17—C14—C15—C16	179.6 (3)
C6—C1—O7—C8	−5.3 (4)	C12—N11—C16—C15	−2.4 (5)
C1—O7—C8—C9	−174.5 (3)	C10—N11—C16—C15	178.4 (3)
O7—C8—C9—C10	62.9 (3)	C14—C15—C16—N11	1.6 (5)
C8—C9—C10—N11	178.4 (2)		

### Hydrogen-bond geometry (Å, º)

**Table:**

*D*—H···*A*	*D*—H	H···*A*	*D*···*A*	*D*—H···*A*
C10—H10*A*···O7	0.97	2.52	2.850 (3)	100
C10—H10*B*···Br1^i^	0.97	2.89	3.735 (3)	146
C10—H10*A*···Br1	0.97	3.11	4.043 (3)	163
C12—H12···Br1	0.93	3.08	3.940 (3)	154
C15—H15···Br1^ii^	0.93	3.07	3.931 (3)	155
C17—H17*B*···Br1^ii^	0.96	3.08	3.956 (4)	152

Symmetry codes: (i) *x*+1/2, −*y*+3/2, −*z*+1; (ii) *x*+1, *y*, *z*.
